# Residual stress distribution analysis of heat treated APS TBC using image based modelling

**DOI:** 10.1016/j.dib.2017.06.016

**Published:** 2017-06-11

**Authors:** Chun Li, Xun Zhang, Ying Chen, James Carr, Simon Jacques, Julia Behnsen, Marco di Michiel, Ping Xiao, Robert Cernik

**Affiliations:** a School of Materials, University of Manchester, Oxford Rd, Manchester M13 9PL, United Kingdom; b ESRF-The European Synchrotron, 71, Avenue des Martyrs, Grenoble, France

## Abstract

We carried out a residual stress distribution analysis in a APS TBC throughout the depth of the coatings. The samples were heat treated at 1150 °C for 190 h and the data analysis used image based modelling based on the real 3D images measured by Computed Tomography (CT). The stress distribution in several 2D slices from the 3D model is included in this paper as well as the stress distribution along several paths shown on the slices. Our analysis can explain the occurrence of the “jump” features near the interface between the top coat and the bond coat. These features in the residual stress distribution trend were measured (as a function of depth) by high-energy synchrotron XRD (as shown in our related research article entitled ‘Understanding the Residual Stress Distribution through the Thickness of Atmosphere Plasma Sprayed (APS) Thermal Barrier Coatings (TBCs) by high energy Synchrotron XRD; Digital Image Correlation (DIC) and Image Based Modelling’) (Li et al., 2017) [1].

## Specifications Table

**Table:** 

Subject area	Materials
More specific subject area	Thermal Barrier Coatings, Residual Stress Analysis
Type of data	Finite Element Analysis (FEA) Result
How data was acquired	Abaqus CAE, Scanip
Data format	TIFF
Experimental factors	Specimen was treated at 1150 °C for 190 h
Experimental features	The microstructure was observed by CT, the achieved image was meshed by Scanip and the residual stress distribution was analysed using FEA by Abaqus CAE
Data source location	Manchester, United Kingdom
Data accessibility	Data is included with this article

## **Value of the data**

• The FEA study of residual stress distribution in APS TBC is based on the real 3D microstructure observed by CT.
• The slices from different positions in the 3D model show the residual stress distribution at the edge of the model as well as inside the model.
• The residual stress along different paths can show the effect of the geometry of the interface between the top coat and the bond coat on the local stress distribution.

## 1 Data

**Fig. 1:**
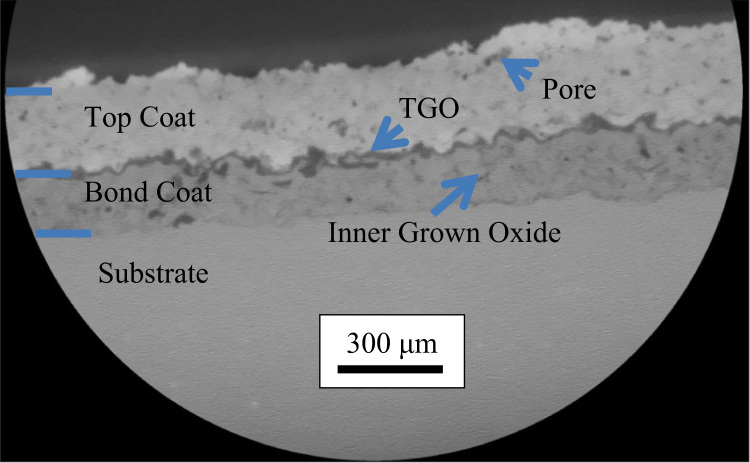
One slice from the 3D microstructure observed by CT, top coat, bond coat, TGO and substrate can be distinguished; some large pores and inner grown oxide can also be observed.

Fig. 1 shows a slice from the 3D microstructure observed by CT; the global 3D microstructure can be found in the related paper [1]. In this slice, top coat, bond coat, TGO and substrate can be distinguished. Some large pores and thick inner grown oxidation can also be observed from the slice.

**Fig. 2:**
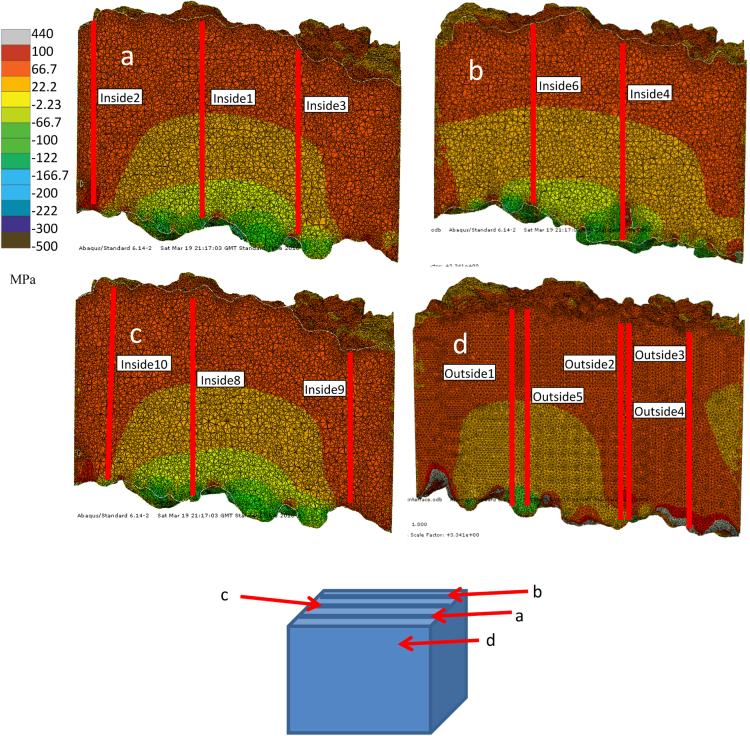
Contour plots of the modelled stress distributions in the top coat of the TBC as a function of position as shown. Plots a–c are inside the 3D model and plot d is on the surface.

Fig. 2(a–d) show the residual stress distribution of the slices inside and the edge of the 3D model, the position of the slices in the 3D models are shown in Fig. 1e. The largest force residual of all the nodes in the model is 2.14×10^−12^ N.

**Fig. 3:**
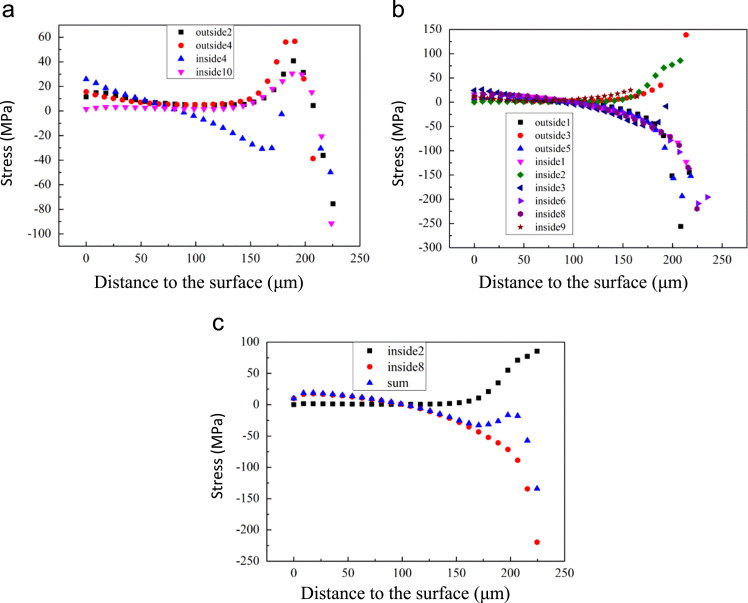
Shows stress distributions calculated along the paths shown in Fig. 1. (a) shows the stress distributions along outside2, outside4, inside4 and inside10, (b) shows outside1, outside3, outside5, inside1, inside2, inside3, inside6, inside8 and inside9, (c) shows the stress distribution along inside2 and inside8 and the summation of those two stress distributions which is similar to the stress distribution in a.

Fig. 3 shows the stress distribution along different paths. Three kinds of residual stress distribution can be observed. The first kind of trend is where the residual stress is compressive, first increasing from the surface to the interface, decreasing a little and then increasing again to the interface. A “jump” feature can be observed about 100–150 μm away from the interface, as shown in Fig. 3(a). The second kind of trend is where the residual stress increases from the surface to the interface; the third kind is where the residual stress first decreases, then becomes tensile and increases to the interface. These two kinds of residual stress trends are shown in Fig. 3(b). The summation of the last two kinds of trend is shown in Fig. 3(c). This is similar to the first kind where a “jump” feature can be observed near the interface.

## 2 Experimental design, materials and methods

The samples were fabricated at the University West [2], [3]. The 7–8 wt% yttria stabilized zirconia (YSZ) top coat and the NiCoCrAlY bond coat were fabricated by APS and the thickness of the top coat and the bond coat was 250 μm and 150 μm, respectively. The substrate was Hastelloy X [4]. The APS TBC is cut into 10 mm×10 mm×~5.5 mm and then heat treated at 1150 °C for 190 h. The microstructure of the APS TBC was observed by CT at the Henry Mosley X-Ray Image Facility (HMXIF) using a Zeiss Xradia versa 520 X-Ray microscope. The measured 3D image was then segmented, and meshed using Scanip. The details of the CT operating parameters, the image processing procedure and the FEA model parameters can be found in the related paper [1]. The mechanical properties of different parts in the model is also shown in the related paper [1]. The image based model was then imported into Abaqus CAE and solved.
