# Supplementary material for: Residual stress distribution analysis of heat treated APS TBC using image based modelling
Source: Data Brief. 2017 Jun 11;13:557–61. doi: 10.1016/j.dib.2017.06.016 (PMC5496202; doi:10.1016/j.dib.2017.06.016)
Supplement: Supplementary file 1 — Supplementary material [file mmc1.doc]

Conflicts of Interest Statement	 	


Manuscript title:  	


The authors whose names are listed immediately below certify that they have NO affiliations with or involvement in any organization or entity with any financial interest (such as honoraria; educational grants; participation in speakers’ bureaus; membership, employment, consultancies, stock ownership, or other equity interest; and expert testimony or patent-licensing arrangements), or non-financial interest (such as personal or professional relationships, affiliations, knowledge or beliefs) in the subject matter or materials discussed in this manuscript.


Author names:


The authors whose names are listed immediately below report the following details of affiliation or involvement in an organization or entity with a financial or non-financial interest in the subject matter or materials discussed in this manuscript. Please specify the nature of the conflict on a separate sheet of paper if the space below is inadequate.


Author names:

This statement is signed by all the authors to indicate agreement that the above information is true and cor- rect (a photocopy of this form may be used if there are more than 10 authors):


Author's name (typed)	Author's signature	Date
